# Crisis and disaster management in the light of the Islamic approach: COVID‐19 pandemic crisis as a model (a qualitative study using the grounded theory)

**DOI:** 10.1002/pa.2217

**Published:** 2020-06-19

**Authors:** Nawal A. Al Eid, Boshra A. Arnout

**Affiliations:** ^1^ Department of Islamic Studies, Faculty of Arts Princess Nourah Bint Abdulrahman University Riyadh Saudi Arabia; ^2^ Department of Psychology King Khalid University Abha Saudi Arabia; ^3^ Department of Psychology Zagazig University Zagazig Egypt

## Abstract

The current study sought to generate a theory from the data on crisis management in Islam, and also aimed to identify the strategies used by leaders in the crisis management process. The grounded theory approach was applied, which is one of the qualitative designs. The content of the verses of the Noble Qur'an and the hadiths of the Prophet Muhammad that dealt with the issue of crises were analyzed. The results of the qualitative analysis of the verses of the Qur'an and the hadiths of the Prophet's noble Sunnah have resulted in four concepts that constitute a broad conceptual theory of crisis management according to the Islamic approach. These concepts are: Crisis management strategies in Islam, the stages of crisis management, the characteristics of a leader who manages crises, and the roles of a leader during the crisis management process. A number of assumptions have been made of this generated theory about Islamic crisis management model. In light of the results of this study, recommendations were formulated that indicate the necessity of training leaders in the Islamic approach to crisis management, its strategies and its scientific steps in crisis management. These results have wide applications in the field of training leaders, and also recommend future studies to verify the assumptions of the theory that emerged from the data in this study on crisis management in Islam (COVID‐19 pandemic crisis as a model).

## 1 INTRODUCTION AND THEORETICAL BACKGROUND

In light of recent events in the 21st century, it is becoming extremely difficult to ignore the existence of wars, crisis, and disasters, that which led us to call the 21st century as a century of crises, given the changes it witnessed in the various political, economic, demographic, health, and environmental aspects of life, including the outbreak of the pandemic of COVID‐19, which affected human life within the social entity, educational, and professional. Indeed, there crises added major challenges for individuals, institutions and decision‐makers, so facing these crises are necessary to avoid more human, material, and moral losses. The crisis is a dangerous and unexpected threat to the goals of individuals, institutions, and nations.

Evidence suggests that the lives of nations and peoples are not without crises that recur throughout the ages. Throughout history, peoples and civilizations have faced adversity at various levels, including what has claimed complete civilizations, including what they have faced until they are gone, and Islamic history abounds with many of the ordeals that the nation has gone through since the dawn of the message and until today, the nation has weakened at times and emerged from it more often in promises. Crisis management depends on the savvy and efficient decision‐makers during these crises. One of the crises that the world is going through today is the COVID‐ 19 pandemic. That has killed hundreds of thousands of individuals around the world, and caused huge economic losses. And it negatively affected the education sector and others, which called upon countries to develop plans to address these losses in all sectors.

Arnout et al. (Arnout et al., 2020, p. 29) mentioned “among the events witnessed in 2020 is the spread of the Corona virus (COVID‐19) by its rapid spread and the increase in the number of infections and the number of deaths in all countries of the world. As a result, the Director‐General of the World Health Organization announced on the eleventh of March 2020 that COVID‐19 represents Pandemic. Given the seriousness of the results of the Corona pandemic (COVID‐19), countries had to intensify their efforts to confront this pandemic, and put plans to manage this crisis to face its severe economic, health, educational, and social consequences. That is why state institutions are keen to use various strategies that emphasize participation among workers in these institutions and all members of society, and collective leadership in thinking and implementation, then follow‐up and evaluation. And activating these strategies through forming committees, work teams, organizing data on crises, preparing and training individuals to face this pandemic and reducing its destructive effects.”

Al Eid, Alqahtani, et al. (Al Eid et al., 2020, p. 1) reported that the Holy Quran and the Sunnah provide the framework for Islamic law (Shareaa), which governs all aspects of life of every Muslim. The Holy Quran is observed as the authority of how to surrender to Allah's will in various circumstances in life. The Sunnah considers to be complemented to the Holy Quran; it could be defined as the teachings and sayings of the Prophet Muhammad.

Recent evidence suggests that the modern crisis management theories have focused on models that include steps for crisis management, including identifying crises and collecting data on them, setting alternatives and choosing from them, then implementing solutions and evaluating their results. Thus, we note that such administrative models ignore some aspects of crisis management in Islam. That is why this study came to generate a broad conceptual theory clarifying crisis management, according to the Islamic model for establishing Islamic crisis management from the Noble Qur'an and the noble Sunnah. This is to take advantage of previous experiences to read and anticipate the future by knowing the past, and on the other hand, highlighting the Islamic model, which was characterized by skill, patience, and faith in crisis management.

### 1.1 Crisis management

The issue of crisis has received considerable critical attention. Bieber (1988) showed that the crisis as a turning point from unstable situations, and could lead to undesirable outcomes, if the parties concerned are unwilling or unable to contain them and ward off their dangers. Mustafa (2005) pointed crisis management is the continuous management process that is concerned with forecasting potential crises by sensing, monitoring internal and external environmental variables generating the crisis, and mobilizing the resource and available capabilities to prevent the crisis or deal with it with the most efficiency and effectiveness, and to achieve the least amount of damage. However, Farid and Ajwa (2005) added that crisis management is a strategic planning process that requires the institution to take a set of decisions in circumstances of tension and uncertainty, at a specific time aimed at the proper response to the events of the crisis and preventing its escalation, and minimizing its negative results to the least possible extent and removing risks in the direction of restore their natural conditions.

In this article, we argue that the crisis is an emergency that damages society and may lead to its collapse, and it may be economic and may be social. Strategies as viewed by the scientists of organization and administration in Islam mean the future vision for any action, task or matter in the future, so that the decision‐maker will have a vision of his command when achieving the destination.

A number of researchers have reported the features of crisis. Barton (2007) determined six features of the crisis, as follows:

1. *Surprise*: It means that crises occur without warning, or ring bells, but rather suddenly.
2. *Lack of information*: This means the lack of information on the cause of this crisis, and the reason is due to the lack of information, especially if it occurs for the first time.
3. *Escalation of events*: when crises occur, juveniles follow to tighten the noose on decision‐makers.
4. *Loss of control*: all events of the crisis fall outside the ability and expectations of the decision‐makers, so they lose control and control.
5. *Panic*: The crisis causes a state of panic, so the decision‐maker will dismiss all those involved in the occurrence of the crisis, or resort to quarrels with his aides.
6. *The absence of a rapid, fundamental solution*: crises do not give the decision‐maker a time or opportunity to reach a careful solution, but rather it is necessary to choose between a limited number of solutions and choose the least harmful.

And through the above, crisis management is a modern concept in the science of management, but crises and dealing with them are as old as man himself, and Islam is the first to start laying the scientific and practical foundations for crisis management, and this was evident through the directives of the Holy Qur'an and honorable hadiths, such as preparing for crises before they occurred, and verification of the validity of information, and strength in facing crises, strategic planning for crises, working in teams to face crises, and strategies to face crises.

### 1.2 The importance of COVID‐19 pandemic crisis management

The world on the planet faces risks from time to time, and the way out is always through science, where technology plays a very important role. Advanced nations seek to confront risks through scientific means and studies, but this time the matter was more dangerous and faster than some people think. It is the new Corona virus, which caused panic among the population of the Earth.

No country in the world has been spared the COVID‐19. This new COVID‐19 has left its effects in all aspects of life; entire states are paralyzed, closed borders, global economies have slowed down, and schools closed. Students in their homes study, employees in their homes work and so on. The spread of epidemics is always a challenge for human societies, such as the new COVID‐19 in 2020, the Spanish flu in 1918 and the black plague in the 14th century over societies. Although Europe in the Middle Ages, after its exit from the First World War, differs greatly from today's world and society in light of the means of communication, the internet, and globalization, the spread of a pandemic is always a test for society and an era. However, the current crisis of the spread of the COVID‐19 pandemic has swept the world, leaving infections in large numbers, and global health systems have collapsed with them. The epidemic threatens social relationships, and caused a change in health systems and produced the concept of quarantine and the creation of methods for sterilization, and all this is undoubtedly the health system, social, educational, family, economic, and leisure are collapsed. The result is the emergence of a new face of this world.

What is clear to everyone now is that a virus that does not see with the naked eye was able to create chaos in this world, and compel everyone to rearrange the vocabulary of his life in proportion to preventive procedures. This pandemic virus, which reached the level of the epidemic, has sounded alarm bells to pay attention to many things, the most important of which is maintaining personal hygiene and adopting a balanced healthy, nutritional, and athletic style. But the inevitable and cruel matter is stopping life, which has its manifestations (closing schools, filling hospitals and clinics, closing clubs and public places, closing some government interests—closing houses of worship), and this virus has turned into an international trend that is spoken by young, old, sick, and health people, and this is indicates the seriousness and ambiguity of the situation, and a description of the state of panic among everyone. From all this, we must manage the COVID‐19 crisis with efficiency and wisdom to reduce the negative effects on the individual and society.

There is a consensus among researchers (Abu Farah, 2009; Abu Khalil, 2001; Al‐Khudairi (2003); Al‐Momani, 2007; Al‐Sheikh, 2008; Fathi, 2002; Maher, 2006; Mustafa, 2005) that crisis management focuses on the use of the scientific method in confronting crises, working to prevent the occurrence of the crisis as possible, and facing the crisis effectively to mitigate its negative effects, loss of lives, and reduce health threats.

Al‐Sheikh (2008) and Al‐Yehoy (2006) mentioned that crisis management is important because of it is providing the scientific ability to extrapolate and predict current and potential threat sources, and optimizing the resources and capabilities available to reduce the effects of a crisis. As well as providing practical capabilities and material capabilities to prepare and confront, and work to return to normalcy through a set of steps and procedures for restoration.

### 1.3 The stages of management crisis

Izz al‐Din (1990) and Maher (2006) determined that there are three phases of crisis management:

1. Pre‐crisis phase: It includes all preventive procedures that avoid the occurrence of the crisis.
2. The stage of dealing with the crisis: it includes all procedures to achieve the maximum possible results.
3. Post‐crisis phase: It includes all the procedures necessary to readapt to the outcome of the crisis, and this adjustment must be achieved in the behavioral, psychological, organizational, and financial aspects.

While Al‐Tayeb (1992) and Bin Abdullah (2003), the crisis management has four stages:

1. The stage of mitigating the crisis, in which the quality of the risks and the surrounding circumstances and the prediction of the surrounding hazards are determined.
2. The stage of preparation and preparation and drawing up a complete plan to face the crisis.
3. The stage of confrontation: It is a decisive stage in managing the crisis, on which the size of the losses depends.
4. The rebalancing stage: in which a relatively long‐term plan is drawn up according to the effects of the crisis.

Thus, the leaders and officials must be aware of these stages to manage the crisis, because it is considered a type of diagnosis, and accordingly, the necessary treatment is determined for these crises. For this, there must be adequate preparations and methods to prevent crises by discovering early warning signals, identifying areas of weakness and addressing them so that they do not turn into a crisis (Ahmed, 2002; Al‐Momani, 2007; El‐Hamlawy, 1995; Maher, 2006; Mustafa, 2005).

By presenting this theoretical framework for crisis management, it is clear that although some research has been carried out on crisis management models, there has been little quantitative analysis of the Islamic approach of crisis management. A search of the literature revealed few studies which attempted to investigate the Islamic crisis management model, thus this study highlight the importance of this object.

## 2 OBJECTIVES

The specific objective of this study was to generate a theory on crisis management according to the Islamic model, as well as formulate their assumptions and to identify the strategies used by leaders in crisis management. And also, to determine the characteristics of the leader during the crisis management process, and the roles assigned to him in this process of crisis management.

## 3 RESEARCH METHODOLOGY

### 3.1 Method

The researchers started from the premise of the explanatory model, as the methodology of the grounded theory that belongs to qualitative research was applied to generate a broad conceptual theory that explains the practical practice of managing crises and disasters according to the Islamic approach. And that is through analyzing the documents of the Noble Qur'an, the noble Sunnah, and by extrapolating the stories of the prophets. As there is a gap between empirical and descriptive studies that dealt with crisis management, clarifying the Islamic model in crisis and disaster management and developing an integrated approach that benefits decision‐makers and reduces the size of heavy losses on the other hand. That is why the current study came to bridge this gap using the grounded theory approach, which, as Glaser and Strauss (1967) pointed out that the aims of the grounded theory method to reach a theory emerged from the data collected in an inductive way.

### 3.2 Participants

According to Glaser and Strauss (1967), the theoretical samples in this study were taken during the data collection process from the texts of the Noble Qur'an, hadiths from the Prophet's Sunnah, and the stories of the Prophets, in order to generate the theory, by collecting, compiling, and analyzing data at the same time, to gradually crystallize the theory.

### 3.3 Tools

The study applying the content analysis of the texts of the Noble Qur'an, the hadiths of the Prophet's Sunnah, and extrapolating how crises were managed according to the Islamic approach, and the steps that were followed in managing these crises, in order to develop a theory clarifying crisis management in Islam. The texts of the Qur'an and hadiths were collected from the Sunnah of the Prophet, then they were organized and categorized, and a qualitative analysis of their content was conducted on them, extracting topics and answering research questions and presenting the results of the study on crisis management theory according to the Islamic approach from the Book of God and the Sunnah of the Prophet Muhammad.

The researchers in this study provided a rich and detailed description of the study context and method, which allows, as Marshall and Rossman (2006) mentioned the transferability in the qualitative studies means the portability of the findings and interpretations of the data to other similar contexts. Thus, the study readers can transmit the results of this study and benefit from it in other similar context through.

### 3.4 Data analysis

The researchers in this study followed the approach of the grounded theory, and according to this methodology, data was collected and analyzed together at the same time, and the data analysis in this study included organizing, classifying, interpreting, understanding their data, noting the patterns, topics, categories, and axes that appear during the analysis.

The data were organized by creating three volumes, the first contains files of Quranic texts and hadiths of the Prophet's Sunnah that dealt with crisis management. The second volume contains the analytical cues, for each Quranic or hadith of the Sunnah in a special file and coded by printing them and then shading the symbols in different colors, as the notes were coded, and the notes are utilized by their repeated reading, and extracting meanings from them to support the process of interpreting the data and writing the results of the study.

While analyzing the data, the researchers continuously compared coding and categories to discover similarities and differences, so that similar categories are grouped together under a concept or a higher category according to the Glaser and Strauss (1967). The coding and comparison process took place after an analysis of every Quranic and hadith of the Prophet's Sunnah, so that these symbols are essential and guided when analyzing the rest of the Qur'anic texts and Hadiths of the Prophet's Sunnah, and this procedure is known as the theoretical samples which, as Charmaz (2014a) indicated, is a feature of the grounded theory methodology as it helps in building categories and themes that refine the theory emerging from the data.

The researchers in this study continued in this way until reaching the stage of theoretical saturation, which means not only the absence of new information in the data, but also the confirmation of concepts that were clearly and accurately identified to construct the theory according to Corbin and Strauss (2008).

**FIGURE 1:**
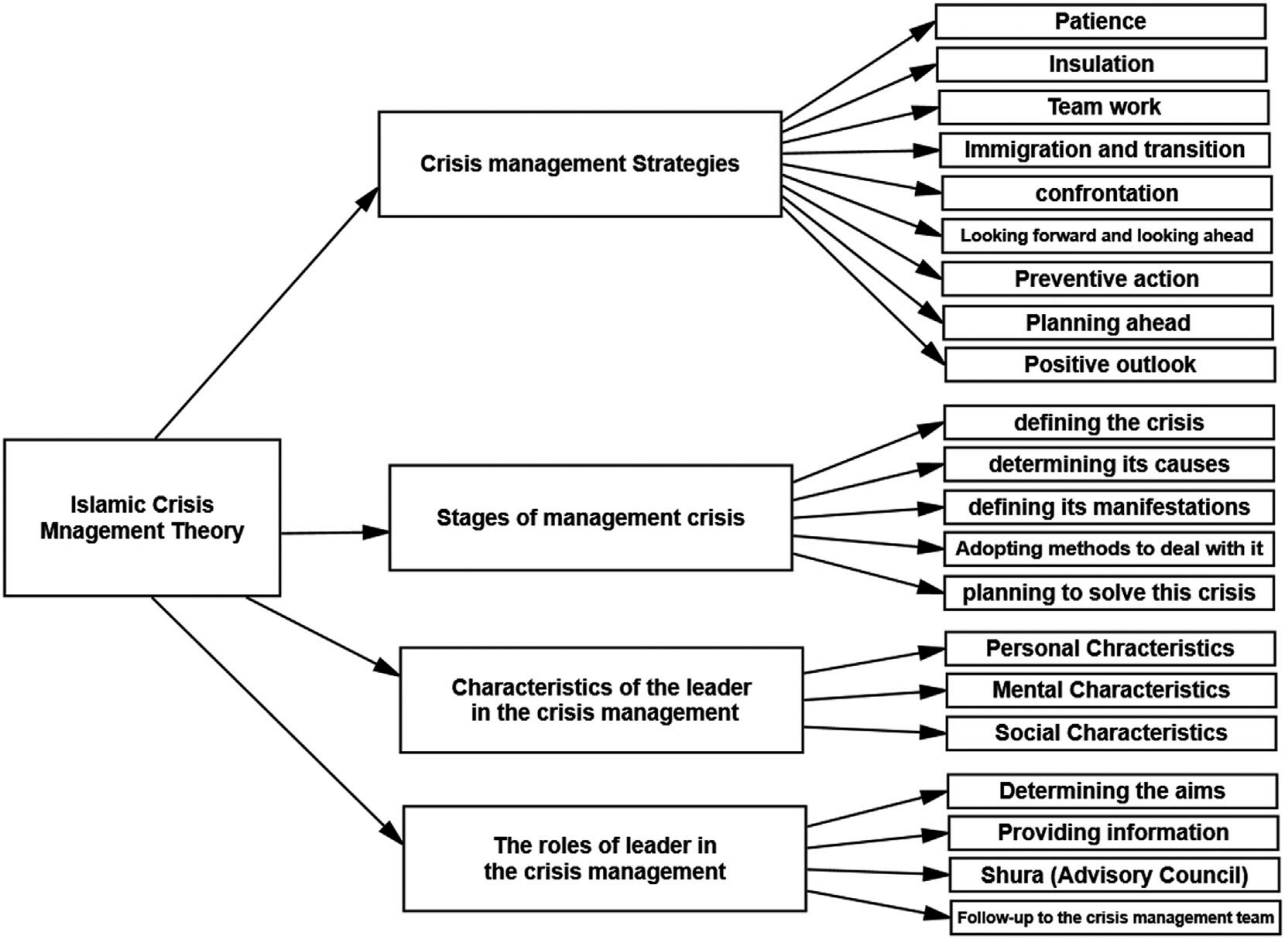
Selective coding of axial groups

In this study, the researchers employed Corbin and Strauss (2008) strategy in data analysis and coding, which includes three coding stages: open coding, axial coding, and selective coding. The open coding resulted in (860) an initial symbol, then it was reduced to the axial coding, its intensification and its aggregation in higher categories through continuous comparison, and establishing links between the groups that emerged from the open coding. In this axial coding, attention is paid to symbols that have a greater analytical value and are repeated and are closely related to research questions. Also, symbols that were not repeated during the analysis process and have nothing to do with research questions were excluded. From the axial coding, 12 basic concepts were identified. After the features of theory began to emerge, which represent the climax of axial coding, the beginning of selective coding, and classification under a basic category (Corbin & Strauss, 2008). In selective coding, the basic concept of the theory is generated and linked in a theory that may help, according to Glaser (1978), in the conceptual integration of crisis management theory in Islam. Figure 1 illustrates the selective coding of axial groups.

## 4 RESULTS AND DISCUSSION

**FIGURE 2:**
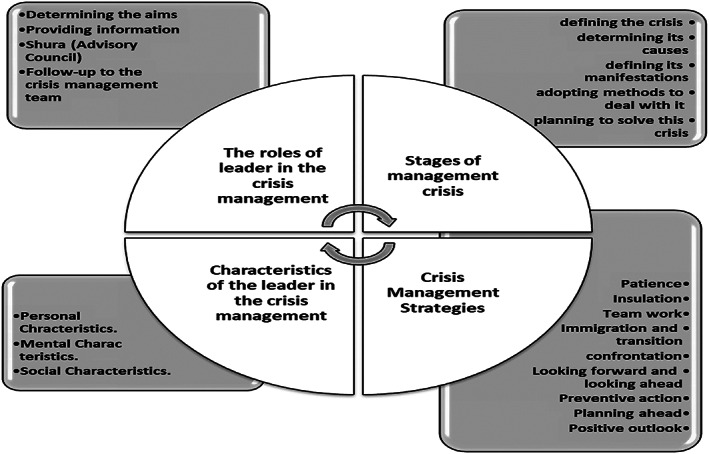
Islam crisis theory, emerging from data

The process of analyzing and coding data revealed four main axes that constitute the theory of crisis management in Islam: stages of crisis management, crisis management strategies, characteristics of a leader in crisis management, tasks in crisis management. Each of these axes contains a number of categories. Figure 2 illustrates the findings of the data analysis and coding process and the resulting theory.

### 4.1 Stages of crisis management

Through the analysis of the Qur'anic texts and prophetic hadiths, it becomes clear to us that the crisis is an emergency situation that harms society and may lead to its collapse, and it is based on economic and social types. Islam has developed an approach for coping crises characterized by wisdom and positivity, and therefore the nation has not weakened or collapsed. This Islamic approach is to manage crises in several steps that are: defining the crisis and determining its causes, then defining its manifestations, then adopting methods to deal with it and planning to solve this crisis.

In the story of the Prophet Noah—peace be upon him—there was a crisis, which was the lack of obedience of his people to him, and the nonentry of new believers in the Islamic religion. The manifestations of this crisis were the severe mockery of the believing group, stubbornness and refusal to hear from the Prophet, no matter how he was exposed in the morning or evening, and insisted on stubbornness and arrogance, so that they would be sedition and delusion for those who actually believed.

As for the method of the prophet Noah's management of this crisis, he began with warning, advice, awareness, reminders, education, and alerting, despite the length of time that Noah broadcast—peace be upon him—calling on his people, but he did not despair, did not retreat, and did not lazily report his call to God by all possible means, to the extent that they put their fingers In their ears, so that they would not hear it, and sought their clothes in order not to see it, so God almighty told him that he would not believe from them except those who believed, he called on them not to be a temptation for the weak believers, so God's punishment for facing the believers was a major crisis, which is the Lord's punishment that will flood the earth with those on it.

In the story of the prophet Noah with his people, when they did not obey him to enter Islam, the reaction of the prophet Noah was patient, did not give up, did not lazy, or retracted his goal in communicating the call of his Lord, and he remained for many years calling them and he only believed with him very little.

### 4.2 Crisis management strategies in Islam

The Islamic nation went through many crises and disasters, among these crises the flood of Prophet Noah who drowned the land, in Surah Al‐Ankabut‐verse‐14 God said “*And We certainly sent Noah to his people, and he remained among them a thousand years minus fifty years, and the flood seized them while they were wrongdoers*” (The Noble Qur'an).

The crisis also including the stormy food crisis that toppled the Arabian Peninsula and the region for seven full years, including the crisis of Muslims during the era of the Prophet Muhammad when they lost safety of themselves and their families, so permission to emigrate, and then the siege crisis unfairness of the faithful in the city from all of the Arabian Peninsula also almost claimed the Islamic nation. In Surah At‐Tawbah‐verse‐40 God “*said If you do not aid the Prophet—Allah has already aided him when those who disbelieved had driven him out [of Makkah] as one of two, when they were in the cave and he said to his companion,*”*Do not grieve; indeed Allah is with us*. “*And Allah sent down his tranquillity upon him and supported him with angels you did not see and made the word of those who disbelieved the lowest, while the word of Allah—that is the highest. And Allah is Exalted in Might and Wise*” (The Noble Qur'an).

And also, by analyzing of the Holy Qur'an verses and the Sunnah of the Prophet Muhammad we found that crises are multiple and varied, and we can divide the crises from an Islamic perspective, as follows:

1. *Economic crises*: such as poverty, unemployment, loans, etc.
2. *Social crises*: including the marriage crisis, divorce, marital infidelity, the housing crisis … and others.
3. *Political military crises*: such as the Battle of Al'ahzab, Talot and Goliath.
4. *Health crises*: such as disease, epidemics, and plague.

Each category of these crises requires different strategies to confront and overcome them. In the story of the Prophet Joseph with his people, a crisis occurred and he expected the scarcity of water after 7 years, the Nile's level decreased for seven whole years, and because the people believed, and the honest and ruled scientist in them, and because they acquiesced to his interpretation and were instructed to set a logical solution, the crisis went well despite the distress. In Surah Yusuf‐Verse‐47 God said “*You will plant for seven years consecutively; and what you harvest leave in its spikes, except a little from which you will eat*.” And also in Yusuf‐verse‐48 “Then will come after that seven difficult [years] which will consume what you saved for them, except a little from which you will store” (The Noble Qur'an).

As well as, among the crises faced by the Prophet Mohammad at the beginning of the message and the call of the people to the Islamic religion, when the few believers threatened to perish and then destroy the invitation or repel those who fear it, so the solutions were limited to leaving the place of infidelity and fleeing to a better country that this group can save itself in order to preserve the debt it holds. In surah Al‐ Tawbah‐verse‐40 God said “*If you do not aid the Prophet—Allah has already aided him when those who disbelieved had driven him out [of Makkah] as one of two, when they were in the cave and he said to his companion,*”*Do not grieve; indeed Allah is with us*. “*And Allah sent down his tranquillity upon him and supported him with angels you did not see and made the word of those who disbelieved the lowest, while the word of Allah—that is the highest. And Allah is Exalted in Might and Wise*” (The Noble Qur'an).

The decision to emigrate was not easy for the people of Mecca, who settled in cities and countries other than the nomadic Arabs who used to move from place to place. Beginning from the first day of the Prophet Muhammad's migration to Medina and crises followed over the nascent society, the first crisis was to provide a place for housing, food and drink for the people of Mecca, migrants leaving behind their homes, money, family and trade, so he took my brother among the migrants and supporters after the souls were prepared to compete for giving and giving at the same time that In it, immigrants refrained from accepting money for free from work and making an effort. In Surah An‐Nisa‐verse‐100 God said “*And whoever emigrates for the cause of Allah will find on the earth many [alternative] locations and abundance. And whoever leaves his home as an emigrant to Allah and His Messenger and then death overtakes him—his reward has already become incumbent upon Allah. And Allah is ever Forgiving and Merciful*” (The Noble Qur'an).

The crisis management strategies of the Prophet Muhammad did not differ completely from the Islamic method in crisis management. One of the basic solutions to confront poverty and the deteriorating economic conditions is to enable those able to work by providing them with job opportunities. Like what happened in the migration crisis, as it was necessary to find a suitable place in the prevailing work of immigrants, which is trading at that time, and to provide a market in which Muslim merchants and workers are active, and Islam has been keen since its foundations in Medina focused on this, in Surah An‐Nisa‐ verse‐100 “*And whoever emigrates for the cause of Allah will find on the earth many [alternative] locations and abundance. And whoever leaves his home as an emigrant to Allah and His Messenger and then death overtakes him—his reward has already become incumbent upon Allah. And Allah is ever Forgiving and Merciful*” (The Noble Qur'an).

We conclude from this that the Prophet Mohammad's wise approach to crisis management was to focus on the main goal, and not to get involved in side events, in Surah Al‐Ahzab‐verse‐21 God said “*There has certainly been for you in the Messenger of Allah an excellent pattern for anyone whose hope is in Allah and the Last Day and [who] remembers Allah often*” (The Noble Qur'an). In order to ensure crisis management in the Prophet's approach, it is necessary to define the rules and procedures that should be dealt with.

Consequently, the elements for the crisis‐resolution strategy were realized, which is to provide the necessary information on the foundations of dealing with the crisis, its care and its compassion to the parties to the crisis. It is also noted that his superiority, may God bless him and grant him peace, in solving the crisis through methods that the science of management did not know, except in our time, by emptying the crisis from its content, since a crisis cannot be implemented in the organization and lead to the effect of fundamental effects if there was no agreement between the forces of the crisis on their content.

The Prophet Muhammad, also excelled in transforming the crisis path by absorbing its results, recognizing its causes, then overcoming it and addressing its results, as the crisis was transformed from negative to positive. His biography, peace be upon him, is a revelation of life in its various aspects, devotional and cooperative, at the individual, family and group levels, in laying the foundations of the state and in enacting its laws politically, economically and socially.

Through a qualitative analysis of the sayings of the Prophet Muhammad, and his prophetic secrecy in managing economic, social and health crises, it becomes clear to us that the wise leadership of the Prophet Muhammad represented the cornerstone of the success of the establishment of the Islamic state, and how not and he does not speak of passion, in addition to his keenness and mercy on his subjects, which inspired him to finding strategies to solve crises that management scientists did not know until today.

From this we deduce that crisis management strategies in the Islamic model include the following:

#### 4.2.1 Patience

God asked his servants with patience when the calamity and the great reward of the patient. God asked the Prophet Muhammad for patience as a strategy for managing crises that he faced in communicating the call to Islam, such as in Surah Al‐Muzzamil—verse 10, God said “*And be patient over what they say and avoid them with gracious avoidance*” (The Noble Qur'an). Patience with enemies and open ends is one of the effective strategies used by the Prophet Muhammad. The psychological factor is the most dangerous when confronting crises and disasters, so the Holy Qur'an was keen to address souls and prove them in times of adversity and afflictions, and to train them that these crises will inevitably end, and therefore we must commit to patience with these disturbances, as feelings of trust, faith, fantasies, and suspicions are controlled. Without this patience and confidence in God's ability, in Surah Al‐Hadid‐verse‐22, God said “*No disaster strikes upon the earth or among yourselves except that it is in a register before We bring it into being—indeed that, for Allah, is easy*” (The Noble Qur'an).

When we trust in God and His ability, we will have the courage to face crises, and therefore crises cannot be overcome, As in the verse 200 in Surah All 'Imran that God said “*O you who have believed, persevere and endure and remain stationed and fear Allah that you may be successful*.” And also, in Al‐Baqarah‐verse‐155‐God said “*And We will surely test you with something of fear and hunger and a loss of wealth and lives and fruits, but give good tidings to the patient*” (The Noble Qur'an).

#### 4.2.2 Insulation

The Prophet Muhammad, peace and blessings be upon him, had established the principles of quarantine a 1400 years and more ago, when he, peace and blessings of God be upon him, said: “If you heard about it on a land, do not step on it.” From the plague like a desert from crooping, and patience with it is like a cricket in crawling Insulation or exclusion is the set of precautionary actions that are used to protect the person from pathogens, meaning that we prevent the arrival or transmission of pathogens to and from the person, such as Narrated Abu Huraira (rad): Allah's Messenger said: “*When one of you wakes up from his sleep, he must not put his hand in a utensil till he washed it three times, for he does not know where his hand was (while he slept)*” (Al‐Asqalani, 2014). The Prophet Muhammad was the first to suggest quarantine and personal hygiene in cases of a pandemic, quarantine means isolating infected people in a specific place and for a specific time with the availability of comprehensive health care until the disease and pathogens are controlled and controlled. This is what the Prophet Muhammad said*, narrated Abu Huraira* “*(There is) no 'Adwa (no contagious disease is conveyed without Allah's permission). nor is there any bad omen (from birds), nor is there any Hamah, nor is there any bad omen in the month of Safar, and one should run away from the leper as one runs away from a lion*” (Al‐Bukhari, 2007). As well as, Prophet Muhammad said: “*If you hear of its presence (the presence of plague) in a land, don't enter it, but if it spreads in the land where you are, don't fly from it*” (Bin Al‐Hajjaj, 2006). In these noble hadiths, an elaborate medical plan developed by the illiterate prophet at a time when there was no one known as a “quarantine” or others, obliging the Muslim who is present in a country where the plague was rampant not to come out of it even if it is healthy because it may have carried the disease, and whoever is outside the country should not enter it.

Also, isolation and blocking epidemics are required from a religious point of view. Islam forbade the exit of a person from an endemic environment into a safe environment, and he does not enter into an endemic environment while in a healthy environment.

#### 4.2.3 Teamwork

From the analysis of the Holy Qur'an we noticed that the Islam urged to cooperate among individuals to work to solve the problems and crises that societies can face. In Surah Al‐ Ma'Idah‐verse‐2 God said “*And cooperate in righteousness and piety, but do not cooperate in sin and aggression. And fear Allah; indeed, Allah is severe in penalty*” (The Noble Qur'an). The importance of forming a crisis management team is evident in the story of Bilqis, the Queen of kingdom Sheba, when she asked her people to let her know about her confrontation with the Prophet Soliman, as in Surah An‐Naml‐verse‐32 “*She said, ‘O eminent ones, advise me in my affair*. *I would not decide a matter until you witness [for] me’*” (The Noble Qur'an).

The idea of forming teams and using them to solve organizational problems and reach results are one of the smartest ideas and trends that smart leaders can use in the best and lasting achievements, in Surah Al‐Kahf‐verse‐95 God said “*He said,That in which my Lord has established me is better [than what you offer], but assist me with strength; I will make between you and them a dam’*” (The Noble Qur'an). In Surah Al‐Kahf, he directed the king of Zulqarnain when he made the people of the village feel that this is their village and that they were the first to preserve it and repel any attack and from any party whatsoever. This dam, even if they leave, has given them the reasons for strength, which is how to form work teams, and once again give them the reasons for strength, which is building the dam and their ability to maintain their village, and therefore they can stay and continue.

Abu Farah (2009) mentioned that to prepare for any crisis, a crisis management team is required to be formed to face any crisis of any kind and it is called the work team, the investigation committee, the expert group, the problem‐solving committee or the special committee, and in the political and military field it is called like the Special Operations Forces or the rapid deployment forces.” The goal of the team is to work to address the crisis, reduce its seriousness and negative effects, in addition to the presence of the leadership of the crisis, that is, advance planning and develop future scenarios to predict any crisis that could arise through the presence of indications of its occurrence.

This crisis management team must be distinguished by the ability to successfully intervene in crisis management, whether it is physical, mental, or practical abilities. This is in addition to composing, commitment to orders and instructions, whatever the risks involved in the crisis, extreme attention when carrying out a task, as well as preparing to sacrifice, loyalty and full membership in the administrative entity (Fathi, 2002).

#### 4.2.4 Immigration and transition

Migration and transmit from one place to another at the time of crises and disasters that threaten people's lives is a strategy to cope these crises. Immigration was a strategic solution for Muslims in the century of Prophet Muhammad to rid themselves of persecution and genocide, after God had asked the Prophet Muhammad to migrate from Mecca to Medina. In Surah An‐Nisa‐verse‐97 God said “*Indeed, those whom the angels take [in death] while wronging themselves—[the angels] will say,*”*In what [condition] were you? They will say,*”*We were oppressed in the land*. “*The angels will say,*”*Was not the earth of Allah spacious [enough] for you to emigrate therein?* “*For those, their refuge is Hell—and evil it is as a destination*.” *As well as, it was narrated from Kathir bin Murrah that Abu Fatimah told him that he said: O Messenger of Allah, tell me of an action that I may do and persist in it*.” *The Messenger of Allah said to him: You should emigrate, for there is nothing like it*” (The Noble Qur'an).

Immigration means moving from one place to another, temporarily or permanently, in residency, whether the migration took place by one person or by a group of people. Migration may be as follows: migration between continents, or migration between countries within a specific continent, or regional migration (within the country itself), and the most important forms of migration are migration from the countryside to cities in search of better life opportunities, and the search for work, and affect Human migration on population, social, cultural and economic patterns and characteristics in the new country.

#### 4.2.5 Confrontation

Managing the crisis by transforming risks, misfortune and threats into opportunities that can be used to maximize the value of the organization. The state's success in managing the crisis requires not allowing this crisis to interfere with the state's regular business, and not allowing these regular actions to interfere with the proposed solutions to the crisis. At this stage, the plan should be put into practice, and the crisis management team should be given the full powers necessary to deal with this crisis. The Crisis Management team should confront the crisis by try more than one method in the event that a method fails and the method depends on solid analytical foundations rather than random methods Prophet Joseph faced the economic crisis by putting the scientific plan out of it by paying attention to agriculture, increasing productivity, reducing losses and storage safety, and rationalizing consumption and surplus. In Surah Yusuf‐verse‐48 God said “*Then will come after that seven difficult [years] which will consume what you saved for them, except a little from which you will store*” (The Noble Qur'an).

#### 4.2.6 Looking forward and looking ahead

It refers to the leadership of the crisis and putting it under control, it also means the direct exit from the problem circle in the solution circle. Also, it means resolving the crises that people or organizations face, which are always ready to deal and predict what happens, and put in their calculations what is not expected to happen through readiness and preparedness, coming out with the least losses and turning the crisis into an opportunity of success in the sense of turning pain into hope, and it needs to exist basic features, skills, and capabilities of the leader of the crisis that distinguishes him from others, including early detection, the prediction of something that turns into a crisis, and also needs self‐confidence and highlighting aspects of strength, validation, and not rushing to judgment and positivity, and optimism, since the presence of crisis leadership prevents the existence and spread of the crisis from during early forecasting and the presence of precursors warning of the crisis, such as, in Surah Yusuf‐verse‐49 God said “*Then will come after that a year in which the people will be given rain and in which they will press [olives and grapes]*” (The Noble Qur'an).

In the Noble Qur'an, God Almighty has given us many examples in the field of prediction, in which there is a prediction of what may happen in the future, with study and analysis of past and present events. Perhaps one of the clearest and most prominent examples in the Holy Qur'an is the story of Moses, peace be upon him, with the righteous servant, the greenness, peace be upon him, which God gave him wisdom and knowledge. In Surah Al‐Kahf‐verse‐78 [Al‐Khidhr] said, “*This is parting between me and you. I will inform you of the interpretation of that about which you could not have patience*” (The Noble Qur'an).

From these results, we can conclude that during crisis management, leaders must avoid indiscriminate confrontation with crises, and they must follow the scientific method, use the initiative management method, pre‐planning to confront them, and define the roles of workers, to reduce the negative effects of the crisis, and benefit from the results of the current crisis for future preventive planning.

#### 4.2.7 Preventive action

Adopting preventive forecasting as a prerequisite in the process of managing crises through proactive management, which is a department based on warning predictive thought to avoid a crisis early by formulating an acceptable preventive system that relies on initiative and innovation and training workers on it. This hypothesis validates that the nature and levels of preparedness in an state vis‐à‐vis crises are directly proportional to the reality of preventive or curative trends among workers in that state. Where the proportional proportionality between the preventive solution to crises and the ability to face crises has been developed at a high level of preparedness. The need to develop and implement preventive and curative awareness programs and training for workers in the field of crisis management on these programs. For example, the locust problem and its destructive effect, which could turn into a seasonal crisis, As in the Yusuf‐verse‐47 [Joseph] said, “*You will plant for seven years consecutively; and what you harvest leave in its spikes, except a little from which you will eat*” (The Noble Qur'an).

And also, When Al‐Khidr, peace be upon him, were accompanied by Moses, peace be upon him, on their journey, they came on the seashore to cross it, and they found a ship that transported people owned by the poor and orphans they used to feed from. And for free. On their way, they were marching in the sea, an unjust tyrant king, who seized every good ship and included it to his navy fleet, so his work was exactly like a sea pirate. Thus, information was available on the past and present of our master, Al‐Khidr, peace be upon him, so he studied and analyzed it, as he knew beforehand about the unjust king, and what he had done with other ships in the past, and he also learned about the situation of the ship of the poor, where their ship was good and valid, and also knew that they would pass on their way in the region of the unjust king assumed the hypotheses, and all indications were that the king would take the ship by force, so he took the decision in this regard to avoid this danger, as he broke the ship and then patched it with another piece of wood, even if the pirates came, they saw this defect and left it to its poor owners, in Al‐Kahf‐verse‐71 God said “*So they set out, until when they had embarked on the ship, al‐Khidhr tore it open*.” *[Moses] said,Have you torn it open to drown its people? You have certainly done a grave thing*” (The Noble Qur'an).

Indeed, had it not been for this future prediction, the ship's owners would have lost their ship, with the unjust seizure of it by the unjust king, so our master Green Plan was in the face of this wise plan. Consequently, we conclude the importance of making plans, and the necessity of predicting the conditions that may occur in the future, relying on the largest possible amount of information and facts, in order to gain the greatest amount of good and pay the greatest amount of evil, first and foremost, all with the permission of God Almighty.

#### 4.2.8 Planning ahead

We note from the analysis of the texts of the Qur'an that Islam as an intermediate religion and belief was able to deal with crises and disasters in an intermediate way as well, as it focused on purely material aspects such as preparation, planning, preparation of materials and work teams and all of these matters that cannot solve the crisis or disaster from others, and besides that parallel to this material aspect, seeking asylum to God. Islam urged for planning in times of crises and disasters, as in Al‐Anfal‐verse‐60 God say “*And prepare against them whatever you are able of power and of steeds of war by which you may terrify the enemy of Allah and your enemy and others besides them whom you do not know [but] whom Allah knows. And whatever you spend in the cause of Allah will be fully repaid to you, and you will not be wronged*” (The Noble Qur'an).

Islamic thought preceded modern administrative thought in many steps and rules that were established to deal with crises and disasters. These steps are illustrated by evidence of the legitimacy of preventive planning. Islam focused on proactive planning, in the sense that we plan and put forward proactive plans in a phase known as no disaster or no crisis, so it is always better to imagine—as an individual or as a country—that each crisis—any kind of crisis or disaster—can happen and draw the necessary scenarios that you think will contribute in one way or another to dealing with this crisis if they occur, and can put more than one scenario, if this does not work then it can work the other scenario (Abu Khalil, 2001; Hilal, 2004).

Planning is an important prerequisite in the crisis management process, as our actions are nothing but a reaction and a variation between the random reaction and the planned reaction. Most crises are aggravated because they are human and administrative errors that occurred due to the absence of the organizational base for planning. If we do not have plans to face crises, crises will end itself the way you want it to be, not the way we want it. Through the foregoing, it becomes clear to us that training in crisis planning is one of the basic axioms in successful organizations, as it contributes to preventing the occurrence of the crisis or mitigating its effects and avoiding the element of surprises accompanying it. Also it becomes clear to us that planning gives the crisis management team the ability to conduct an organized and effective response to a crisis with high efficiency, ready to face the unplanned emergency situations that may accompany the crisis.

The biography of our Prophet Muhammad is full of attitudes indicating good planning and management, disguising from the enemy, the ability to overcome all difficulties, reaching the goal, the wise mind, and a correct opinion. And all of these attributes were combined in the greatest migration that history has ever known, the migration of the Prophet Muhammad, peace and blessings be upon him, from Makkah Al‐Mukarramah to Medina, which the Prophet Muhammad had planned well. In the end, from these findings we can say the crisis management is refer to Setting effective plans to manage the crisis, and these plans are important for the organization's success in dealing with this crisis and getting out of it with the least possible negative effects. Where the Prophet guides us to plan for the future, so that prosperity flies over future generations, and that our children and grandchildren live with prosperity, even if it is simple, in order not to be dependent on people, and they ask people other than them. Among those hadiths is the planning dialogue that took place between Saad bin Abi Waqas and the Prophet Muhammad. Narrated Sa'd: “*I became seriously ill at Mecca and the Prophet came to visit me. I said,*”*O Allah's Messenger! I shall leave behind me a good fortune, but my heir is my only daughter; shall I bequeath two third of my property to be spent in charity and leave one third (for my heir)? He said,No*.” *I said,Shall I bequeath half and leave half?*” *He said,* “*No*.” *I said,* “*Shall I bequeath one third and leave two thirds?*” *He said,* “*One third is alright, though even one third is too much*.” *Then he placed his hand on his forehead and passed it over my face and abdomen and said,O Allah! Cure Sa'd and complete his emigration*.” “*I feel as if I have been feeling the coldness of his hand on my liver ever since*” (Al‐Bukhari, 2007).

#### 4.2.9 Positive outlook

A Muslim must not view the crisis as all evil, as a negative view impedes proper thinking that facilitates the attainment of an appropriate solution, in surah Al‐Baqarah‐verse‐216 “*Fighting has been enjoined upon you while it is hateful to you. But perhaps you hate a thing and it is good for you; and perhaps you love a thing and it is bad for you. And Allah Knows, while you know not*” (The Noble Qur'an).

Islam is a religion of optimism and hope, for crises do not increase sincere Muslims except by persistence and optimism, as they have increased faith and delivery, it is a secret of the eternity of the message of Islam, and the steadfastness of a united Muslim, this is how we learned our role models, and our beloved Prophet Muhammad, the path of his optimism Prophet Muhammad in crises is filled with vivid patterns and immortal situations.

Optimism has taken a method for it. In the darkest of circumstances and the most severe crises, but it is positive optimism, optimism that leads to work and not to lethargy and laziness, so his optimism teaches us that mere wishes are not enough to be warranted and lulled to reckoning and backwardness, and that impotence and weakness were never a pretext for lethargy and inaction, it was pure his Prophet Muhammad Wondrous is accompanied by strong certainty, firm and sincere determination, his optimism deepens self‐confidence, cultivates hope for a bright future, and calls for hard work, diligence, patience, and perseverance, his optimism stimulates vigor and activity. Optimism accompanied by firmness and certainty, confidence and good belief in the Lord. In Surah yusuf‐verse‐87 God said “*O my sons, go and find out about Joseph and his brother and despair not of relief from Allah. Indeed, no one despairs of relief from Allah except the disbelieving people*” (The Noble Qur'an).

The positive attitude or optimism is the hope that a positive achievement can be achieved despite any obstacles, and a belief in positive results and their expectation, even in the most difficult situations, crises and challenges. instill in positive souls, take responsibility, love of work, patience and perseverance, whatever the difficulties, and accumulate troubles. And in conclusion: it suffices if not an omen, it will be pessimism, despair, despair, deficiency, and flogging, then conflict, classification, division, fighting, and killing. Accidents and crises are circumstances, filled with lessons and sermons. as in Surah All 'Imran–verse 22, God said “*And those who are patient, seeking the countenance of their Lord, and establish prayer and spend from what We have provided for them secretly and publicly and prevent evil with good—those will have the good consequence of [this] home*” (The Noble Qur'an). As well as, *narrated Abu Huraira*: The Prophet Muhammad said, *There is no disease that Allah has created, except that He also has created its treatment*” (Al‐Bukhari, 2007).

These findings consisted with what Al‐Araji and Dasamsa (2000) and Al‐Khudairi (2003) indicated that efficient crisis management requires strategic planning, prediction and the creation of a trained team for crisis management and teamwork, providing a good information base and system for communications, analyzing past crises to benefit from them as a previous experience, and setting scenarios for facing crises.

### 4.3 Characteristics of leader in crisis management

The results of analyzing the contents of the verses of the Noble Qur'an, and the hadiths of the Prophet Muhammad, showed that the leader who manages the crisis must have several characteristics, we divided these characteristics, as follows:

#### 4.3.1 Personal characteristics

1. Trust in God.
2. Control the crises in which it is facing.
3. Strength in the face of adversity and distress.
4. Honesty.
5. Patience and hardship.
6. Good values, ethics, and behaviors.
7. Optimism and the promise of pessimism.
8. Do not hesitate, firm, and work.
9. Spirituality.

#### 4.3.2 Mental characteristics

1. Knowledge and experience.
2. Intelligence.
3. Promptitude.
4. Thinking skills and the ability to solve problems.

#### 4.3.3 Social characteristics

1. Charisma and ability to influence.
2. Effective communication skills.
3. Building positive relationships with others and gain their trust.
4. Involving the parties to the crisis in taking decisions to solve them and choosing between alternatives.
5. Courage when confrontation.
6. Altruism and goodness to others.
7. Cooperation and support for others.

Today's world is witnessing large numbers and many forms of crises, the extent of which varies between those that occur at the individual level, and those that affect groups of different organizations, whether at the local or global level. And given that the circumstances surrounding us are unstable and successive changes, this has led to the complexity of the crises and their multiple dimensions. Therefore, these crises must be confronted and managed wisely, because their persistence leads to enormous human and economic losses. This crisis management needs distinct and creative leadership, which has personal, mental, and social characteristics and characteristics that enable it to effectively confront these crises.

Al Eid, Arnout, and Almoied, 2020, p. 4) found that the leader must possess the ability to distinguish between reality and imagination and see things as they are without distortion, the ability to excel and transcendence, the ability to engage in deep spiritual states of thinking such as meditation, reverence, intuition, the ability to transcendence above materialism, maximize the experiences of daily work life which enables the leader to understanding his relationships with all beings.

There is no doubt that these characteristics are essential components of crisis management, enabling the leader during crisis management to assess the crisis situation, and analyze the elements of this situation, scientific planning for intervention to the crisis, and also makes him able to intervene to management the crisis.

### 4.4 The roles of leader in the crisis management

The results of analyzing the contents of the verses of the Noble Qur'an and the hadiths of the Prophet Muhammad showed that the leader has roles that he must perform in times of crises and disasters, in order for him to overcome these crises, and the following are the results of devising these roles:

1. *Determining the aims*: How is the path of the search for solutions if there are no clear and specific aims that must be reached, the identification of means is based on setting aims first, and it works without an aim or goal with which all results are equal, and when the directions are equal, there will be no access. It has been narrated on the authority of Umar b. al‐Khattab that the Messenger of Allah said: “*(The value of) an action depends on the intention behind it. A man will be rewarded only for what he intended. The emigration of one who emigrates for the sake of Allah and His Messenger is for the sake of Allah and His Messenger; and the emigration of one who emigrates for gaining a worldly advantage or for marrying a woman is for what he has emigrated*” (Bin Al‐Hajjaj, 2006).

2. *Providing information*: With accurate information, the aim is easily reached in the most direct way. For example, if you want to solve a crisis like the one facing the world today COVID‐19 pandemic, how can you make a decision for a region or country without first knowing information about how the disease has spread, information about its seriousness, information about neighboring countries and the extent of their spread, information about safety methods, information about the economic situation of the people that run them, information about the food stock and how much time is sufficient, information about people's habits and traditions and their response and awareness, here you can make decisions about isolation, media awareness, or home campaigns. Or by preventing flight, to provide financial assistance to groups of the poor countries. In Surah Al‐Kahf‐verse‐78 [Al‐Khidhr] said, “*This is parting between me and you. I will inform you of the interpretation of that about which you could not have patience*” (The Noble Qur'an).

3. *Shura*: The decision‐maker who is influenced by the decision may succeed for some time, but at the time of setback he bore the results alone. Therefore the decision‐maker must take the decision by raises the matter to the Shura, puts everyone in the face of responsibility and chooses the best opinions and does not offend the opinions of his employees (Al‐Momani, 2007; Maher, 2006). In the Battle of the “alkhandaq,” the role of the Leader of the Prophet Muhammad in consulting his soldiers is evident in every small and large as long as there is no Quranic text to do so. Before the army of infidels arrived to return without a fight after a period of severe siege. In Surah Ash‐Shurra‐verse‐38 God said “*And those who have responded to their lord and established prayer and whose affair is [determined by] consultation among themselves, and from what We have provided them, they spend*” (The Noble Qur'an).

The Messenger of God—may God's prayers and peace be upon him—is a good example in applying the principle of Shura and committing himself to it, and his Sunnah included wonderful pictures of the Messenger's consultation with his companions. The Messenger consulted his companions during the Great Battle of Badr, in the exit of the Battle of Uhud, and in the peace of Ghaftan on the trench and other positions of the Prophet Muhammad with his companions. Such as, “*Narrated Abu 'Ubaidah: That 'Abdullah said:On the Day of Badr when the captives were gathered, the Messenger of Allah said: 'What do you (people) say about these captives?*” *Then he mentioned the story in the lengthy Hadith. [Abu 'Eisa said:] There are narrations of this topic from 'Umar, Abu Ayyub, Anas, and Abu Hurairah This Hadith is Hasan, and Abu 'Ubaidah did not hear from his father. It has been reported that Abu Hurairah said:None was more apt to seek council of his Companions than the Messenger of Allah*” (Al‐Tirmidhi, 1996).

4. *Follow‐up to the crisis management team*: This is the role of the leader who assigned the roles but does not leave them to circumstances, but rather adopts the principle of accurate follow‐up by setting interim goals. The determinant, and finishes one quarter of it in the first 2 days of the week, and so on. He set interim goals for accurate follow‐up that would facilitate reaching the final results on time, or at least a large percentage of them. In Surah At‐Tawbah‐verse‐105 God said “*And say,Do [as you will], for Allah will see your deeds, and [so, will] His Messenger and the believers. And you will be returned to the Knower of the unseen and the witnessed, and He will inform you of what you used to do’*” (The Noble Qur'an).

From this we can say that an effective leader in the times of crisis and disasters is one who gives the opportunity to others fully to express their opinions, listen to them well, and does not boast of his incredible mental capabilities, in order to achieve collective interests in overcoming these crises.

From these findings, the theory that was generated from the data collected, about the Islamic crisis management model (see Figure 2), we can formulate these assumptions:

1. Traditional and indiscriminate methods in crisis management do not fit with the nature and complexity of contemporary crises, and therefore it is necessary to follow a scientific method in crisis management that is characterized by complementary, cunning and organization, which is the approach developed by Islam since more than 14 centuries ago.
2. Success in crisis management needs the leader to acknowledge its existence and not to deny it, because this increases the losses incurred by it, and makes it more difficult to face them.
3. The crises differ in their type, severity, and causes, and for this, the strategies for management with them differ.
4. The aims of crisis management are to stop losses and consequential damage, control the movement and eliminate it, take preventive procedures to prevent its recurrence in the future, and forecast future crises.
5. The strategy used for crisis management varies with the crisis stage.
6. Effective crisis management has requirements, including the personal, mental, and social characteristics of the leader who manages these crises.
7. During a crisis management leader, there are roles. The success of crisis management effectiveness depends on the degree of the leaders' awareness and their fulfillment of these roles.

Therefore, these assumptions need future studies to verify them. This is an invitation to researchers specialized in the field of crisis management.

## 5 CONCLUSION

The present study aimed to build a broad conceptual theory by applying the grounded theory approach to the Islamic approach to crisis and disaster management, the strategies used in managing it, the stages of crisis management, the characteristics of the leader who manages crises, and the roles assigned to overcome these crises such as the outbreak of the pandemic COVID‐19. The qualitative analysis of the verses of the Noble Qur'an and the noble hadiths of the Prophet, it is possible to derive the Qur'anic approach from the Book of God and the Noble Prophet's Sunnah. By these results, the study offers some important insights which fills a gap in the literature of the Islamic crisis management model. Therefore, these findings may benefit officials and decision‐makers in crisis management—a pandemic outbreak crisis, COVID‐19—as a model, and also provides them with strategies that enable them to manage these crises effectively to overcome them and eliminate the dire consequences that may result from them, as well as increase their awareness of the stages of crisis management according to the approach Quranic and prophetic, and their insight into the characteristics of the leader who manages crises, and the roles assigned to them. These results may be useful through a qualitative study of crisis management in developing programs to train leaders in crisis management.

There are several important areas where this study makes an original contribution to the Islamic model of crisis management, thus the results of this study may constitute a basis for future studies as a set of basic data, and as a starting point for training leaders in crisis management strategies and stages.

### 5.1 Limitations

Despite the applied importance of the results of the current study, the reader should bear in mind that there are a number of limitations to this study, including the size of the verses of the Qur'an and the noble hadiths were analyzed. The second of these limitations, that is this study consider a qualitative study that applied the grounded theory approach. Therefore, we invite future studies to apply other methods for qualitative research in the study of the topic of crisis management in Islam, such as the case study and the phenomenological study approaches. The third limitation is that the results of this study should not be considered widely general, but researchers can use these results presented to them as a baseline and hypotheses for other qualitative and quantitative studies on the topic of crisis management.

## 6 RECOMMENDATIONS

In light of the results of this study, we recommended that:

1. Training leaders in the crisis management strategies.
2. Training leaders in the crisis management stages process.
3. Developing leaders' awareness of the characteristics of leaders' managing crisis.
4. Increasing leaders' awareness of the roles required of them when managing crises.

## CONFLICT OF INTEREST

The authors declare no conflicts of interest.

## ETHICS STATEMENT

The authors of this manuscript have complied with ethical principles in their treatment of individuals participating in the research policy described in the manuscript.

## Data Availability

All data underpin this study were found in this manuscript, there are no any additional data.
